# Role of gamma-giardin in ventral disc formation of *Giardia lamblia*

**DOI:** 10.1186/s13071-019-3478-8

**Published:** 2019-05-14

**Authors:** Juri Kim, Soon-Jung Park

**Affiliations:** Department of Environmental Medical Biology and Institute of Tropical Medicine, Yonsei University College of Medicine, Seoul, South Korea

**Keywords:** *Giardia lamblia*, Gamma-giardin, Ventral disc, Cell-cycle control

## Abstract

**Background:**

*Giardia lamblia*, a protozoan pathogen causing diarrheal outbreaks, has characteristic cytoskeletal structures including eight flagella, a median body and a ventral disc. Gamma-giardin is a unique component protein of the cytoskeleton of this protozoan.

**Results:**

Through comparative proteomic analysis between different stages of the cell cycle, *G. lamblia* γ-giardin (Glγ-giardin) was identified as an upregulated protein in the G2-phase. Increased Glγ-giardin expression in G2 was confirmed by western blot and real-time polymerase chain reaction analyses. Knockdown of this protein using a morpholino affected the formation of ventral discs, especially the microribbons of the discs, but exerted little effect on the binding ability of *G. lamblia*. The number of cells with four nuclei was increased in Glγ-giardin-knockdown cells. Expression of Glγ-giardin was decreased during encystation, in contrast with the G2-phase.

**Conclusions:**

Knockdown experiments demonstrated that Glγ-giardin is a component of the trilaminar structure of the ventral disc. Expression of Glγ-giardin is induced in the G2-phase prior to active cell division, whereas its expression decreases during encystation, a dormant stage of *G. lamblia*.

**Electronic supplementary material:**

The online version of this article (10.1186/s13071-019-3478-8) contains supplementary material, which is available to authorized users.

## Background

*Giardia lamblia* is a protozoan pathogen causing gastrointestinal diseases in humans [1]. Infection is initiated by ingestion of a metabolically dormant and infective form, the cyst, which is converted into trophozoites in the small intestines of the hosts *via* excystation. Trophozoites are the multiplying form responsible for the pathogenesis of giardiasis. Through the encystation process, some trophozoites transform into cysts before being released outside the host.

At present, limited information is available on the mechanism of how a trophozoite divides into two progenies or how the division process is regulated. Based on the finding that the cell cycle of *Giardia* trophozoites can progress despite blocked DNA synthesis, double stranded DNA breaks or defective mitotic spindles, this pathogen has been reported to have defective cell cycle checkpoint systems [2]. Investigations on cell cycle control in *Giardia* trophozoites have been performed primarily by obtaining synchronized cell cultures using aphidicolin [3] or nocodazole/aphidicolin [4]. Attainment of synchronized *Giardia* cultures was improved with the use of counterflow centrifugal elutriation [5]. In this study, *Giardia* cells were prepared as enriched cultures at G1/S and G2 using aphidicolin in order to identify *Giardia* proteins showing phase-specific expression. One of the overexpressed proteins in the G2-phase was identified as γ-giardin, which is a known *Giardia*-specific component of the ventral disc [6].

The *Giardia* trophozoite has unique cytoskeletal structures essential for its survival and pathogenicity, including four pairs of flagella, a median body (MB) and a ventral disc [7]. Microtubules (MTs) composed of α-/β-tubulin are the basic constituent of the *Giardia* cytoskeleton [8]. In addition, a group of proteins between 29 and 38 kDa are known as giardin and have been identified as unique components of the ventral discs [9, 10]. The proteins belonging to the giardins are classified into four subgroups, α-, β-, γ-, and δ-giardins, not related to their amino acid sequences. Alpha-giardins of 33 kDa include annexin showing a phospholipid-binding ability [11], along with 21 putative α-giardin genes found in the *G. lamblia* database [12]. A β-giardin of ~30 kDa contains small coiled-coil segments of four heptads and comprises the ventral disc of *Giardia* trophozoites [13]. A γ-giardin of 38 kDa is also a component of the ventral disc, the microribbon of *Giardia* trophozoites [6]. Delta-giardin, localized in the ventral disc, has been reported to be involved in attachment of *Giardia* trophozoites to the intestinal epithelium [14].

The ventral disc is one of the characteristic structures of the *Giardia* trophozoite that has been the focus of ultrastructural investigation [15]. As an organelle involved in attachment, this structure is located on the ventral aspect of trophozoites, and is formed by spiral layers of MTs wound clockwise around the central bare area. Adjacent to each disc MT, microribbons are associated with the basal MT layer along the full length of MTs. These microribbons are crosslinked with horizontal bridges [8, 16, 17]. Fragmentation and shrinkage of these discs has been reported during cell division and encystation of *G. lamblia* [18, 19]. Analysis of *Giardia* structures using transmission electron microscopy with thin and semi-thin sections and cryo-techniques, and by immunofluorescence microscopy using anti-tubulin antibodies demonstrated that the ventral disc seems to play a role in the division process, participating in karyokinesis [20, 21]. In the present study, the roles of *G. lamblia* γ-giardin (Glγ-giardin) in the ventral disc formation and cell division of *G. lamblia*, was examined by decreasing its expression using a morpholino.

## Methods

### Cultivation of *G. lamblia* trophozoites

Trophozoites of *G. lamblia* WB strain (ATCC30957; American Type Culture Collection, Manassas, VA, USA) were grown for 72 h at 37 °C in TYI-S-33 medium (2% casein digest, 1% yeast extract, 1% glucose, 0.2% NaCl, 0.2% l-cysteine, 0.02% ascorbic acid, 0.2% K_2_HPO_4_, 0.06% KH_2_PO_4_, 10% calf serum and 0.5 mg/ml bovine bile, pH 7.1) [22].

### *Giardia* trophozoite synchronization using aphidicolin and flow cytometry analysis

For synchronization of *Giardia*, aphidicolin (Sigma-Aldrich, St. Louis, MO, USA) at a concentration of 5 μg/ml was added to cells grown to about 60% confluency. Control cells were treated with 0.05% DMSO which was used to solubilize 5 µg/ml aphidicolin. After a 6-h incubation at 37 °C, the medium was replaced with drug-free fresh TYI-S-33 culture medium and incubated for an additional 3 h.

Flow cytometry analysis of various cells (DMSO-treated control, aphidicolin-treated and aphidicolin-washed trophozoites) was performed as previously described [3]. Briefly, the harvested cells were re-suspended in 50 μl of TYI-S-33 culture medium and were treated with 150 μl of cell fixative (1% Triton X-100, 40 mM citric acid, 20 mM dibasic sodium phosphate and 200 mM sucrose, pH 3.0) at room temperature for 5 min. The samples were diluted with 350 μl of diluent buffer [125 mM MgCl_2_ in phosphate-buffered saline (PBS: 137 mM NaCl, 2.7 mM KCl, 10.1 mM Na_2_HPO_4_ and 2 mM KH_2_PO_4_, pH 7.4)] and then stored at 4 °C until use. Fixed cells were reacted with 2.5 μg of RNase A (Sigma-Aldrich) and 10 μg/ml of propidium iodide (Sigma-Aldrich) for 30 min at 37 °C. The cells were evaluated with respect to their DNA content by flow cytometry and FlowJO software analysis (FlowJo Llc, Ashland, OR, USA).

### Two-dimensional gel electrophoresis (2-DGE) of *Giardia* extracts and image analysis

One milligram of protein extracts was prepared from about 3 × 10^8^ trophozoites arrested with aphidicolin (G1/S-phase cells) and trophozoites released from the aphidicolin-mediated arrest (G2-phase cells) by resuspending them in a 2-DGE sample buffer (7 M urea, 2 M thiourea, 100 mM DTT, 4.5% CHAPS and 40 mM Tris). Immobilized pH gradient (IPG) gel strips (pH 3–10, 18 cm; GE Healthcare, Uppsala, Sweden) were soaked overnight with the extracts. The rehydrated IPG strips were treated for sequential isoelectric focusing at 80 kV. Second-dimension separation was carried out at room temperature on 9–17% sodium dodecyl sulfate (SDS)-polyacrylamide gel electrophoresis (PAGE) gels (20 × 25 cm). The protein bands on the gel were visualized by Coomassie staining. The 2-DGE experiment was performed twice. Stained gels were scanned using a GS-710 imaging densitometer (Bio-Rad, Hercules, CA, USA) and analyzed with Melanie 5 image analysis software (GE Healthcare). Image labeling was processed using Adobe Photoshop v.7.0 software. Analysis of these two-dimensional (2-D) gels was performed at Yonsei Proteome Research Center (Seoul, Korea).

### Liquid chromatography mass spectrometry

Protein spots on the 2D gel were excised and digested with trypsin. The trypsin-treated proteins were analyzed by quadrupole time-of-flight (Q-TOF) mass spectrometry (MS) in addition to matrix-assisted laser desorption ionization-TOF MS. Product ion spectra were collected in the information-dependent acquisition mode and were analyzed with an Agilent 6530 accurate-mass Q-TOF MS (Agilent Technologies, Santa Clara, CA, USA). For the Q-TOF liquid chromatography-tandem MS (LC–MS/MS) data sets, tandem mass spectra were submitted to our Mascot in-house database search engine (NCBI NR database downloaded on 31 July 2009). For protein identification, a Mascot ion score of > 55 was used as the criterion for a meaningful result.

### Construction of *G. lamblia* expressing HA epitope-tagged Glγ-giardin

A 936-bp DNA fragment of the Glγ-giardin gene (GiardiaDB ORF no. GL50803_17230) was amplified from *G. lamblia* WB genomic DNA by PCR using two primers, γ-giardin-NcoI-F and γ-giardin-HAX3-R (Table 1). NcoI and NotI sites, located at the ends of the Glγ-giardin DNA fragment, were used for cloning into the corresponding sites of plasmid pGFP.pac [23], resulting in the plasmid, pGlγ-giardinHAX3.pac (Table 2).

**Table 1:** Primers and morpholino used in this study

Name (GiardiaDB ID)	Nucleotide sequence (5′–3′)^a,b^
Transgenic *G. lamblia* expressing HA-tagged Glγ-giardin	
γ-giardin-NcoI-F (GL50803_17230)	CATCCCATGGATGAAGTCATCGTTCTCGACCG
γ-giardin-HAX3-R (GL50308_17230)	GTTACGCGGCCGCTTA*AGCGTAATCTGGAACATCGTATGGGTAAGCGTAATCTGGAACATCGTATGGGTAAGCGTAATCTGGAACATCGTATGGGTA*ATCAACCTTCGTCGTCATGATC
Recombinant *G. lamblia* cyclin B	
3977-F (GL50803_3977)	CCCAAGCTTATGCATTAGACGACGAA
3977-R (GL50803_3977)	CCGCTCGAGCTTTGCTTCCTTTGTATT
Real-time PCR	
γ-girdin-RT-F (GL50803_17230)	GCATCCGAGAGAAACATAAA
γ-giardin-RT-R (GL50803_17230)	TTAATCAACCTTCGTCGTCA
Actin-F (GL50803_15113)	GTCCGTCATACCATCTGTTC
Actin-R (GL50803_15113)	GTTTCCTCCATACCACACG
Mopholino sequences	
Control	CCTCTTACCTCAGTTACAATTTATA
Anti-Glγ-giardin (GL50803_17230)	CAATATAAACGCACATTGCGAAGAG
Anti-GlMBP (GL50803_16343)	GCTGAAAACCATAGCCTCGGACATT

^a^Restriction enzyme sites are underlined

^b^Mutated bases are indicated as italic letters

**Table 2:** Strains and plasmids used in this study

Organism/plasmid	Description	Source/reference
*Giardia lamblia*		
ATCC 30957	Clinical isolate	ATCC
*Escherichia coli*		
DH5α	*supE44*, *ΔlacU169 (Φ80 lacZ ΔM15)*, *hsdR17*, *recA1*, *endA1*, *gyrA96*, *thi-1*, *relA1*	Invitrogen
BL21 (DE3)	*F9*, *ompT*, *hsdSB(rB-mB-) gal*, *dcm (DE3)*	Invitrogen
Plasmids		
pGFP.pac	Shuttle vector, Amp^R^, *pac* gene	[23]
pGlγ-giardinHAX3.pac	pGFP.pac, 936-bp encoding *glγ-giardin* (GiardiaDB ID GL50803_17230)	This study
pET21b	Expression vector, Amp^R^	Novagen
pET-cyclinB	pET21b, 1,026 bp encoding *G. lamblia* cyclin B (GiardiaDB ID GL50803_3977)	This study

*Abbreviations*: Amp, ampicillin; Kan, kanamycin; ^R^, resistant; DNA-BD, DNA binding domain; AD-activation domain; HA, hemagglutinin

The trophozoites were grown for 72 h in TYI-S-33 medium. Thirty micrograms of an episomal plasmid, pGlγ-giardinHAX3.pac, were transfected into 1 × 10^7^ trophozoites by electroporation under the following conditions: 350 V, 1000 μF and 700 Ω (Bio-Rad). Transfection was performed with 5 independent sets of *Giardia* trophozoites at once. Trophozoites harboring the plasmid pGlγ-giardinHAX3.pac were initially selected by adding puromycin (AG Scientific, San Diego, CA, USA) to the TYI-S-33 medium at a final concentration of 10 µg/ml, and further enriched in the medium containing 50 µg/ml of puromycin at 4–5 days post-transfection. We obtained 4 *Giardia* lines showing the puromycin resistance. Among them, two *Giardia* cell lines demonstrated expression of HA-tagged Glγ-giardin as shown in western blot analysis using anti-HA antibodies. *Giardia lamblia* trophozoites carrying pΔ.pac [24] were used as a control.

### Western blot analysis and antibody formation

Cell extracts were prepared from various *G. lamblia* cells (cells without plasmid, cells carrying pGlγ-giardinHAX3.pac, and cells carrying pΔ.pac) in PBS. The extracts were separated by SDS-PAGE and transferred onto a polyvinylidene fluoride (PVDF) membrane (Millipore, Bedford, MA, USA). The membrane was incubated with monoclonal mouse anti-HA (1:1000; Sigma-Aldrich) in a blocking solution [Tris-buffered saline with Tween 20 (TBST): 50 mM Tris-HCl, 5% skim milk and 0.05% Tween 20] at 4 °C overnight. Following incubation with horseradish peroxidase (HRP)-conjugated secondary antibody, immunoreactive proteins were visualized using an enhanced chemiluminescence (ECL) system (Thermo Fisher Scientific, Waltham, MA, USA). Membranes were incubated in a stripping buffer (Thermo Fisher Scientific) at room temperature for 20 min and then reacted with polyclonal rat antibodies specific to the protein disulfide isomerase 1 (PDI1; GiardiaDB ORF no. GL50803_29487) of *G. lamblia* (1:10,000) as loading control. The rat anti-GlPDI1 polyclonal antibodies were obtained by using the recombinant GlPDI1 protein [25].

To make recombinant *G. lamblia* cyclin B (Glcyclin B; GiardiaDB OFR no. GL50803_3977) protein, 3977-F and 3977-R primers (Table 1) were used to amplify a 1026-bp DNA fragment of the *glyclin B* gene. A HindIII and XhoI site were used to clone the PCR products into pET21b (Novagen, Darmstadt, Germany) resulting pET-cyclinB (Table 2). Recombinant Glcyclin B protein was overexpressed in *Escherichia coli* BL21 (DE3) by adding 1 mM isopropylthiogalactoside (IPTG) (Sigma-Aldrich), and then used to make Glcyclin B-specific polyclonal antibodies by immunizing Sprague-Dawley rats (three immunizations at 3-week intervals, 200 μg per immunization). Specificity of polyclonal antibodies against recombinant Glcyclin B was confirmed by reacting them with *E. coli* extracts overexpressing the recombinant Glcyclin B or *Giardia* extract (see Additional file 1: Figure S1).

### Immunofluorescence assay (IFA)

To examine the localization of Glγ-giardin, *G. lamblia* expressing HA-tagged Glγ-giardin was attached to glass slides coated with _L_-lysine in a humidified chamber. The attached cells were fixed with chilled 100% methanol at − 20 °C for 10 min, and permeabilized with PBS/0.5% Triton X-100 for 10 min. After a 1 h-incubation in the blocking buffer (PBS, 5% goat serum and 3% BSA), the cells were reacted overnight with mouse anti-HA antibodies (1:100; Sigma-Aldrich) and rat anti-Glγ-giardin polyclonal antibodies (1:100). Following three 5-min washes with PBS, the cells were incubated with Alexafluor 488-conjugated anti-mouse IgG (1:100; Molecular Probes, Grand Island, NY, USA) and Alexafluor 555-conjugated anti-rat IgG (1:100; Molecular Probes) at 37 °C for 1 h. Slides were mounted with VECTASHIELD anti-fade mounting medium with 4′,6-diamidino-2-phenylindole (DAPI; Vector Laboratories, Burlingame, CA, USA). The slides were then examined with an Axiovert 200 fluorescent microscope (Carl Zeiss, Oberkochen, Germany).

### Quantitative real-time PCR analysis

Total RNA was prepared from G1/S-phase and G2-phase cells using TRizol (Invitrogen, Carlsbad, CA, USA) according to the manufacturer’s instructions. Five micrograms of RNA were converted into complementary DNA (cDNA) using an Improm-II reverse transcription system (Promega, Madison, WI, USA). Real-time PCR was performed using LightCycler System and LightCycler 480 SYBR Green I Master Kit (Roche Applied Science, Mannheim, Germany). Conditions for real-time PCR were as follows: pre-incubation at 95 °C for 5 min followed by 45 amplification cycles of 94 °C for 1 min, 56 °C for 1 min and 72 °C for 1 min. The nucleotide sequences of the forward and reverse primers used for real-time PCR are listed in Table 1. The *G. lamblia* actin-related gene (Glactin; GiardiaDB OFR no. GL50803_15113) transcript was used for normalization of mRNA amount in the cDNA samples.

### Knockdown of Glγ-giardin expression using morpholino

Glγ-giardin expression was decreased using a 25-mer morpholino for the Glγ-giardin open reading frame (ORF) from the start codon (Table 1; Gene Tools Llc, Philomath, OR, USA). The control morpholino, a non-specific oligomer, was used as a control (Table 1). Morpholinos were added to 5 × 10^6^ cells at a final concentration of 100 μM by electroporation. The transfected cells were grown for 48 h, and then analyzed for level of Glγ-giardin by western blot and IFA as described above.

### Scanning electron microscopy (SEM)

Morpholino-treated cells were prefixed in a Karnovsky fixative solution (2% glutaraldehyde, 2% paraformaldehyde, 0.5% CaCl2 in a 0.1 M cacodylate buffer, pH 7.4), followed by washing in 0.1 M cacodylate buffer (pH 7.4) and post-fixing by 1.33% osmium tetroxide in 0.1 M cacodylate buffer (pH 7.4). Thereafter, the samples were dehydrated in absolute ethanol. For observation by SEM, the dehydrated samples were rehydreated with isoamyl alcohol and treated with a 300 Å gold coating using an ion coater (Leica EM ACE600; Leica Microsystems, Vienna, Austria).

### Transmission electron microscopy (TEM)

For transmission electron microscopy, morpholino-treated cells were fixed with 2% glutaraldehyde-2% paraformaldehyde in 0.1 M phosphate buffer (pH 7.4). They were post-fixed with 1% OsO_4_ in 0.1 M phosphate buffer (pH 7.4) for 2 h and dehydrated in an ascending graded series (50–100%) of ethanol and infiltrated with propylene oxide. Specimens were embedded using a Poly/Bed 812 kit (Polysciences Inc., Warrington, PA, USA). After embedding, the specimens were polymerized at 65 °C in an electron microscope oven (TD-700; Dosaka EM, Kyoto, Japan) for 24 h. Next, 70-nm-thin sections were double stained with 6% uranyl acetate and lead citrate (Fisher Scientific, Rockford, IL, USA) for contrast staining. Sections were cut with a Leica EM UC-7 (Leica Microsystems) using a diamond knife and transferred to copper grids. All thin sections were observed with a TEM (JEM-1011; JEOL, Tokyo, Japan) at an acceleration voltage of 80 kV.

### Adherence assay

A total of 5 × 10^6^ *Giardia* trophozoites treated with control morpholino or anti-Glγ-giardin morpholino were cultured in TYI-S-33 medium. After 48 h of cultivation, the culture medium was discarded to remove the non-attached cells and replaced with PBS. The tubes were incubated on ice for 20 min to detach the adherent cells. The detached cells were harvested by centrifugation at 3000× *rpm* for 15 min at 4 °C. The number of cells per milliliter was determined using a haemocytometer.

As a control, knockdown of expression of *G. lamblia* median body binding protein (GlMBP; GiardiaDB ORF no. GL50803_16343) [26] was performed by transfecting anti-GlMBP morpholino (Table 1) into *G. lamblia* trophozoites. Adherence of the resulting transfectants was also monitored as described above.

### Determination of population of cell stage and mitotic index of *G. lamblia* trophozoites

*Giardia* cells treated with control, or anti-Glγ-giardin morpholino, were stained with Giemsa, and the percentage of cells in various stages, i.e. interphase, mitosis, cytokinesis, was then determined. The cells were attached onto glass slides, air-dried, and then fixed with 100% methanol for 10 min. They were then stained with 10% Giemsa solution for 40 min and washed with distilled water. After mounting with dibutyl phthalate xylene (Sigma-Aldrich), the slides were observed with an Axiovert 200 microscope (Carl Zeiss).

At 48 h post-treatment with control morpholino, or anti-Glγ-giardin morpholino, the ratio of cells with two nuclei to those with four nuclei was determined to monitor mitosis, as previously described [27]. The fixed cells were mounted in VECTASHIELD anti-fade mounting medium with DAPI (Vector Laboratories). The numbers of cells with four nuclei or two nuclei were counted in a total of over 300 cells per each condition.

### *In vitro* encystation of *Giardi*a trophozoites

To induce encystation *in vitro*, trophozoites were transferred into an encystation medium (TYI-S-33 medium with 10 mg/ml bovine bile, pH 7.8) [28]. At various time-points after incubation in the encystation medium, cells were harvested by centrifugation at 3000× *rpm* for 15 min at 4 °C. Intracellular levels of Glγ-giardin were determined using anti-Glγ-giardin antibodies. To monitor the encystation process, the intracellular level of cyst wall protein 1 (CWP1) [29] was measured in the harvested *G. lamblia* by western blot analysis using anti-GlCWP1 antibodies [30]. The amount of GlPDI1 was monitored as a loading control. The localization of Glγ-giardin in encysting cells was also observed by IFA as described above.

### Statistical analysis

Data are presented as the mean ± standard deviation from three independent experiments. To determine the statistical significance of these results, data were analyzed using paired samples t-tests. Differences with *P*-values of less than 0.05 were considered significant. In the figures and tables, two asterisks indicate *P*-values of less than 0.01, while a single asterisk indicates *P*-values between 0.01 and 0.05.

## Results

### Identification of γ-giardin as a growth phase-controlled protein in *G. lamblia*

**Fig. 1:**
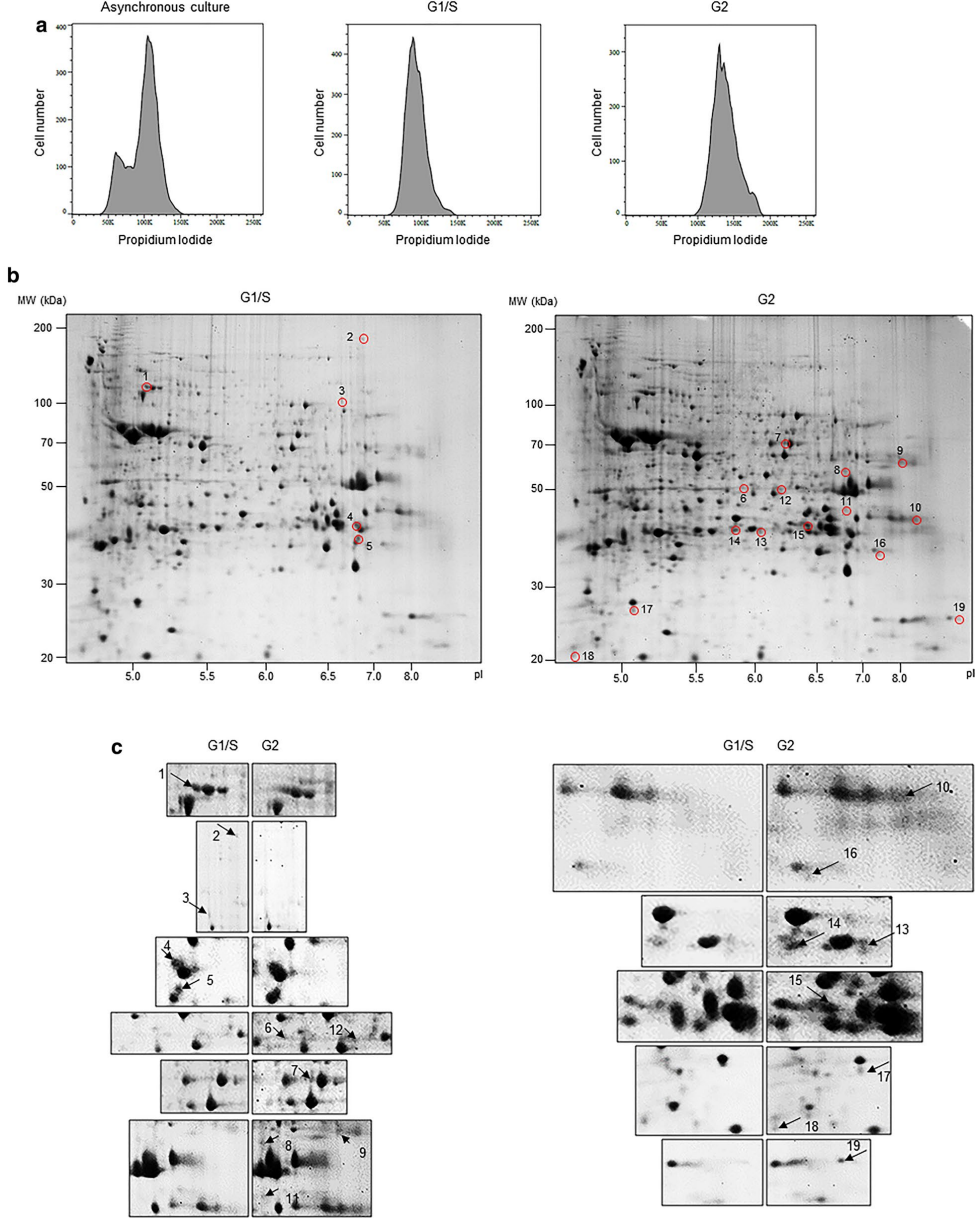
Synchronization of *Giardia* trophozoites using aphidicolin and proteomic analysis of the synchronized cells. **a** FACS analysis of various *G. lamblia* cells (0.05% DMSO-treated cells, 5 μg/ml of aphidicolin-treated cells, and cells released from aphidicolin arrest) on their DNA content using propidium iodide staining. The experiment was performed three times, and three replicates were analyzed for each experiment. **b** Comparative 2-dimensional gel electrophoresis of lysates prepared either from trophozoite arrested at G1/S-phase with aphidicolin or trophozoites at G2-phase released from aphidicolin arrest. Five protein spots (#1 to #5) present only at G1/S-phase and 14 protein spots (#6 to #19) found only at G2-phase, were analyzed by LC/MS-MS; identities of these proteins are listed in Table 3. **c** Parallel comparison of selected protein spots between G1/S- and G2-phase cells in extended panels

To obtain *Giardia* cells at specific stage of cell division, aphidicolin was used. Flow cytometry analysis of the resulting *Giardia* cells indicated that 6-h treatment with aphidicolin produced *Giardia* cells at G1/S-phase, and 3-h release from aphidicolin treatment resulted in *Giardia* cells at G2-phase (Fig. 1a). Control cells, i.e. *Giardia* trophozoites treated with 0.05% DMSO, were found to be a mixture of G1/S-, and G2-phase cells, and the cells at G2-phase were dominant (76%), as reported previously (Fig. 1a) [27].

*Giardia* extracts enriched at G1/S and G2 cells were compared using 2-DGE (Fig. 1b). The 2-DGE experiment was performed twice. Through gel image analysis, we obtained 180 non-pairing spots (50 spots in G1/S-gel and 130 spots in G2-gel), which could be detected only in one of the two samples. Among of them, five spots were selected as upregulated proteins at G1/S-phase whereas 14 spots were chosen as upregulated proteins at G2-phase. Comparison of these spots at G1/S-phase *versus* G2-phase are presented as extended panels in Fig. 1c. Differential expression of these 19 proteins at G1/S-phase *versus* G2-phase was observed in two independent 2-DGE experiments. The identities of these protein spots were investigated with LC–MS/MS analysis (Table 3).

**Table 3:** Identification proteins by 2D analysis

	GiardiaDB ID	Protein name^a^	MW (kDa)^a^	pI^a^	Sequence coverage (%) ^b^	Matched peptide number^b^	Expected value^c^
1	GL50803_88765	Cytosolic HSP70	72	5.26	36	20	7.6e−014
2	GL50803_17063	Pyruvate-flavodoxin oxidoreductase	133	6.41	22	25	1.9e−011
3	GL50803_10521	Arginyl-tRNA synthetase	71	6.48	23	14	0.0022
4	GL50803_9008	Aldose reductase	35	6.61	29	9	0.0022
5	GL50803_16717	Hypothetical protein	29	6.54	44	11	1.2e−005
6	GL50803_11118	Enolase	49	5.46	23	9	0.052
7	GL50803_137703	Protein 21.1	57	5.97	28	15	0.0071
8	GL50803_16124	TCP-1 chaperonin subunit eta	66	7.5	26	15	0.047
9	GL50803_14404	Phosphatase	43	6.14	34	10	0.00039
10	GL50803_21942	NADP-specific glutamate dehydrogenase	50	7.98	51	26	4.8e−018
11	GL50803_16453	Carbamate kinase	34	8.03	44	16	3e−009
12	GL50803_103713	Protein disulfide isomerase 4	41	6.13	35	11	0.00024
13	GL50803_103373	alpha-7.1 giardin	44	8.81	28	12	0.0001
14	GL50803_11043	Fructose-bisphosphate aldolase	35	6.19	34	9	0.028
15	GL50803_17230	Gamma giardin	36	6.48	50	21	0.003
16	GL50803_13273	Dynein light intermediate chain	27	7.68	35	11	0.0012
17	GL50803_17090	*Giardia* trophozoite antigen 1	21	5.05	56	18	1.9e−011
18	GL50803_5333	Calmodulin	17	4.29	66	7	0.00048
19	GL50803_6171	Hypothetical protein	17	9.12	47	8	0.059

^a^Data from 2D gel

^b^Matching masses from LC–MS/MS when searching against all entries in NCBI using Mascot search engine

^c^Probability value obtained from Mascot search

Three of the G1/S-phase upregulated proteins were metabolic enzymes, pyruvate-flavodoxin oxidoreductase, aldose reductase, and arginyl-tRNA synthetase. Cytosolic heat shock protein 70 (HSP70) was found to be increased at G1/S phase of *G. lamblia*. The other one was denoted as a hypothetical protein.

With respect to the G2-phase upregulated proteins, six encoded metabolic enzymes, i.e. enolase, phosphatase, NADP glutamate dehydrogenase, carbamate kinase, protein disulfide isomerase 4 and fructose-bis-phosphate aldolase. Four proteins showing increased expression at the G2-phase were associated with the *G. lamblia* cytoskeleton (α-7.1-giardin, γ-giardin, calmodulin and dynein light intermediate chain). In addition, expression of *Giardia* trophozoite antigen 1 was increased at the G2-phase. No functional information could be obtained for another two clones (protein 21.1 and hypothetical protein) showing increased expression in the G2-phase. Identification of the protein folding related proteins (T complex protein-1 chaperonin subunit eta and HSP70 as an upregulated protein in the G2-phase and G1/S-phase, respectively), was probably caused from the side-effect of aphidicolin [31].

**Fig. 2:**
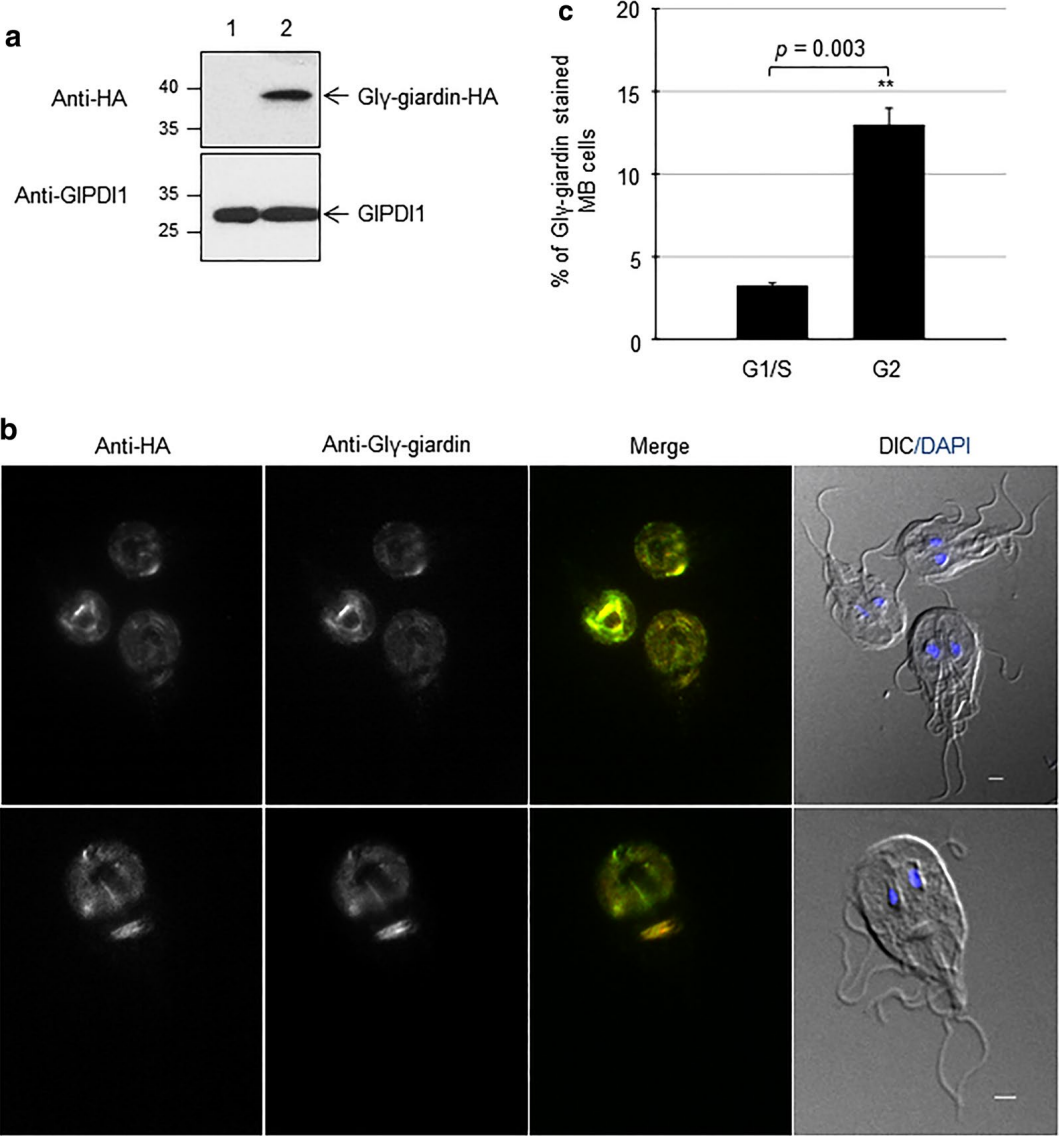
Construction of transgenic *Giardia* trophozoites expressing HA-tagged Glγ-giardin and localization of HA-tagged Glγ-giardin. **a** Expression of HA-tagged Glγ-giardin was confirmed by western blot analysis. Extracts were prepared from *G. lamblia* containing pΔ.pac (Lane 1), or pGlγ-giardinHAX3.pac (Lane 2). Membrane was reacted with monoclonal mouse anti-HA (1:1000). After deprobing antibodies with stripping buffer, membrane was incubated with polyclonal rat antibodies specific to PDI1 of *G. lamblia* (1:10,000) as loading control. **b** Localization of Glγ-giardin in *G. lamblia* expressing HA-tagged Glγ-giardin. *Giardia lamblia* expressing HA-tagged Glγ-giardin attached to glass slides were reacted overnight with mouse anti-HA (1:100) and anti-Glγ-giardin (1:100) then incubated with Alexafluor 488-conjugated anti-mouse IgG (1:100) and Alexafluor 555-conjugated anti-rat IgG (1:100). DIC image was acquired to show cell morphology. *Scale-bars*: 2 μm. **c** Percentages of *Giardia* cells with stained MB at G1/S- and G2-phases

Among the identified protein spots, γ-giardin, a unique cytoskeletal component of *G. lamblia* [6], was selected for further investigation. *Giardia lamblia* γ-giardin, Glγ-giardin, was previously found as a protein interacting with end-binding protein 1, one of microtubule binding proteins [32]. The intracellular location of Glγ-giardin in *Giardia* trophozoites was observed using transgenic *Giardia* cells expressing the HA-tagged Glγ-giardin. A transgenic *Giardia* expressing HA-tagged Glγ-giardin was constructed by transfecting the episomal plasmid, pGlγ-giardinHAX3.pac into *Giardia* trophozoites. Expression of HA-tagged Glγ-giardin was confirmed by western blot analysis using anti-HA antibodies (Fig. 2a). An immunoreactive protein of ~38  kDa was detected in the *Giardia* extracts with pGlγ-giardinHAX3.pac, whereas the protein was absent in extracts of *Giardia* cells carrying the vector plasmid. Localization of Glγ-giardin was examined in the transgenic *Giardia* using anti-HA antibodies and anti-Glγ-giardin antibodies (Fig. 2b). Glγ-giardin was found on the adhesive disc and the MB in double-stained *Giardia* trophozoites. To examine whether localization of Glγ-giardin in MB occurs in a phase-specific manner, percentages of cells with stained MB were measured for both G1/S- and G2-arrested cells. Percentages of *Giardia* cells with stained MB were significantly increased up to 13% at G2-phase from 3.2% at G1/S phase-cells (Fig. 2c).

**Fig. 3:**
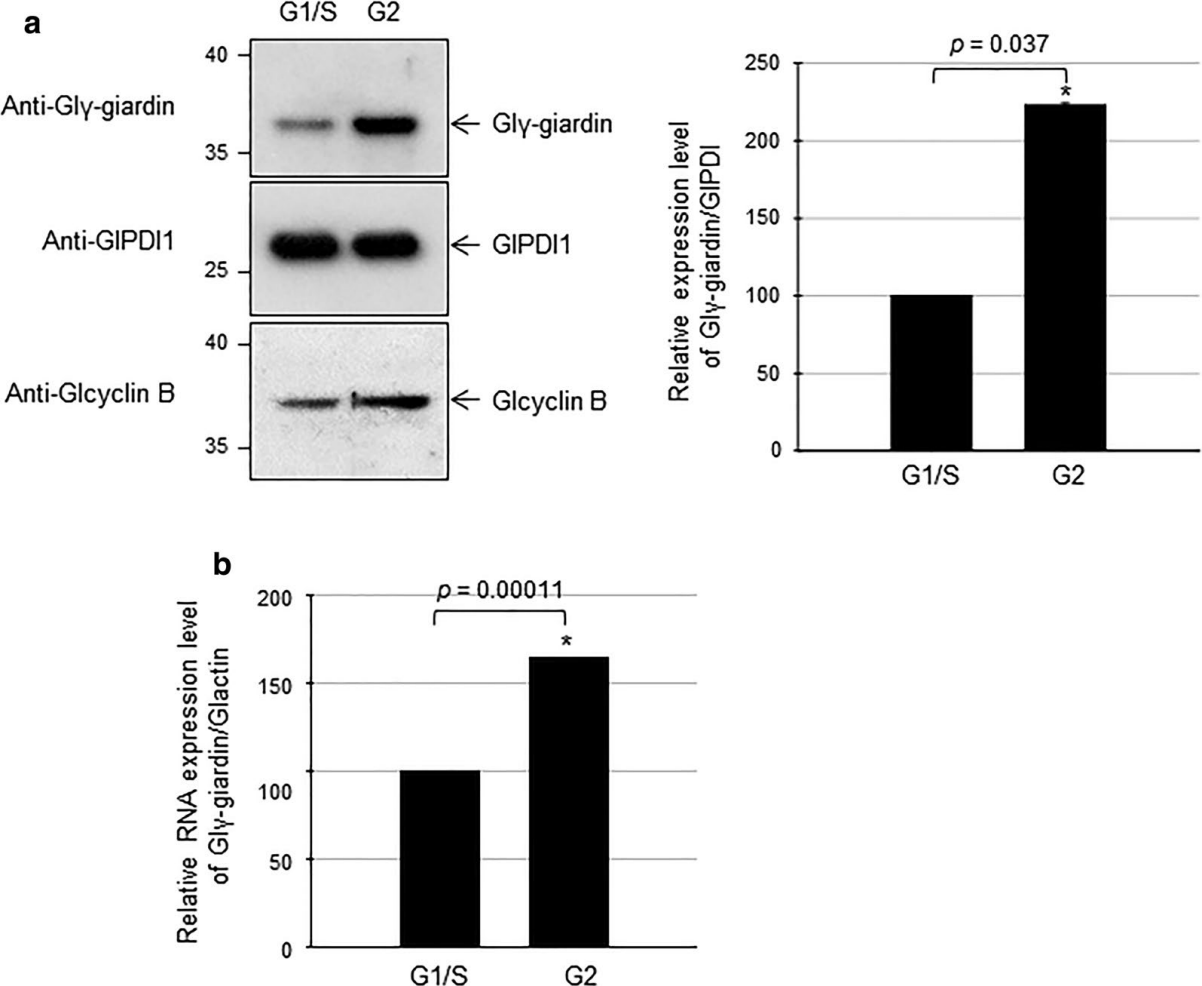
Expression of Glγ-giardin in *Giardia* trophozoites at G1/S- and G2-phases. **a** *Giardia lamblia* were arrested at G1/S phase with aphidicolin (Lane 1) or trophozoites at G2-phase released from aphidicolin arrest (Lane 2). Expression of Glγ-giardin was confirmed by western blot analysis. Membrane was reacted with rat anti-Glγ-giardin polyclonal antibodies (1:1000). After deprobing antibodies with stripping buffer, membrane was incubated with polyclonal rat antibodies specific to PDI1 of *G. lamblia* (1:10,000) as loading control. The same blot was also reacted with rat anti-Glcyclin B polyclonal antibodies (1:500). **b** Total RNA was prepared from G1/S-phase and G2-phase cells using TRizol. Five micrograms of RNA were converted into cDNA using an Improm-II reverse transcription system. Real-time PCR was performed using a LightCycler System and LightCycler 480 SYBR Green I Master Kit. Conditions for real-time PCR were as follows: pre-incubation at 95 °C for 5 min followed by 45 amplification cycles of 94 °C for 1 min, 56 °C for 1 min and 72 °C for 1 min. Nucleotide sequences of forward and reverse primers used for real-time PCR are listed in Table 1. Normalization of mRNA quantity in the cDNA samples was performed using *glactin* transcript levels

Increased expression of Glγ-giardin in the G2-phase was examined by western blot analysis (Fig. 3a). *Giardia lamblia* WB trophozoites were used to make G1/S-arrested cells, and G2-arrested cells as described above. Western blot analysis of these extracts using anti-Glγ-giardin antibodies demonstrated a 2.2-fold increase in the protein level of Glγ-giardin at G2-phase in comparison with the G1/S-phase cells. Western blot analysis of the same blot using anti-GlPDI1 antibodies served as a loading control [25]. These G1/S- and G2-phase cells were analyzed for levels of *G. lamblia* cyclin B (Glcyclin B; GiardiaDB ORF no. GL50803_3977), which is a G2-phase marker in *G. lamblia* [33] (Fig. 3a). The amount of Glcyclin B was increased 1.8-fold in the G2-phase cells, indicating that our samples were arrested at G1/S- and G2-phase by aphidicolin.

Upregulation of Glγ-giardin was also examined with an alternative method, quantitative RT-PCR (Fig. 3b). The level of Glγ-giardin transcript at G2-phase was 1.6-fold higher than that of G1/S-phase cells.

### Effect of knockdown of Glγ-giardin on cell division of *G. lambla*

**Fig. 4:**
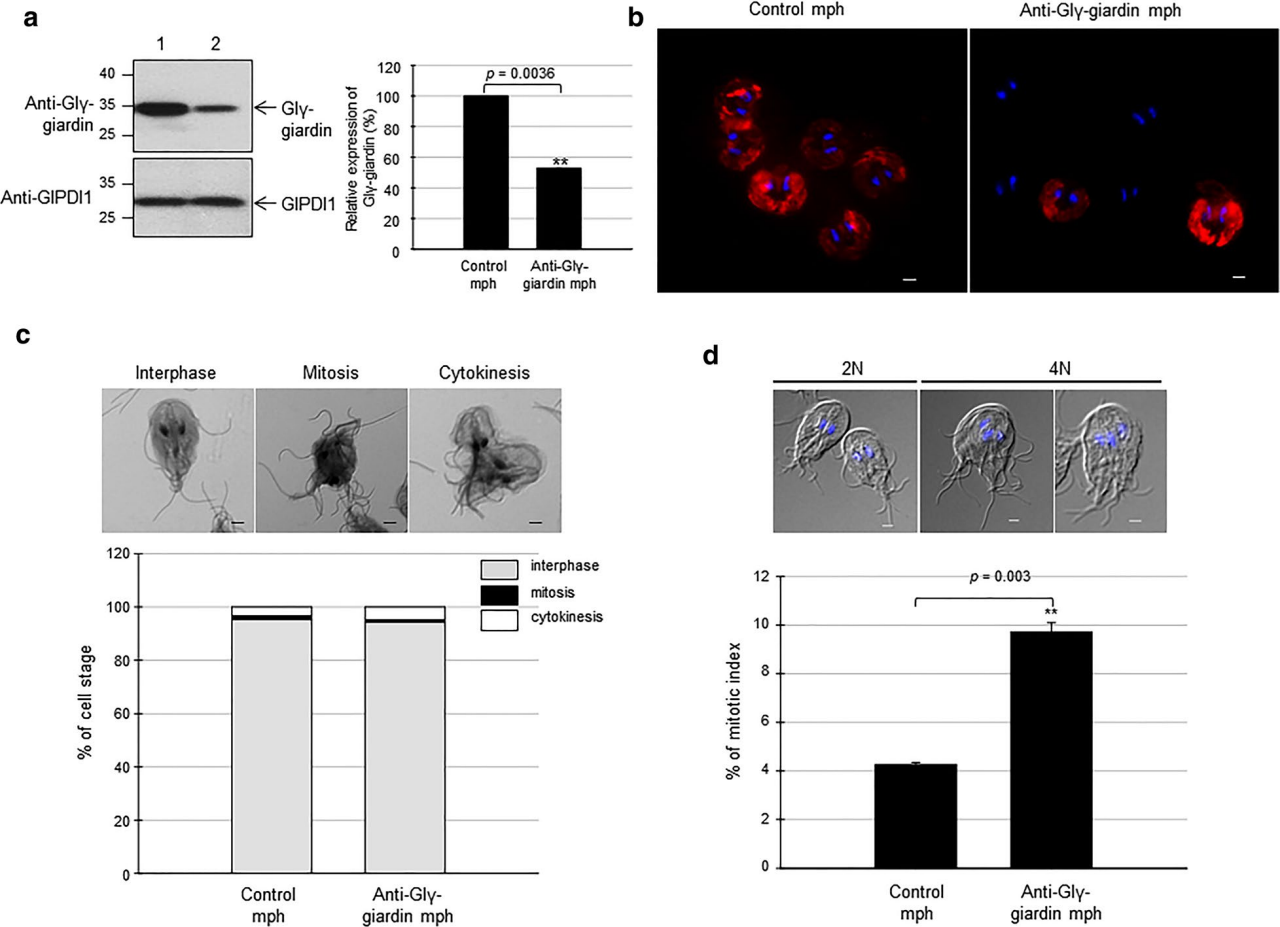
Effect of morpholino-mediated knockdown of Glγ-giardin on *Giardia* cell division. **a** *Giardia* trophozoites were collected 48 h after electroporation with control morpholino (control mph; Lane 1), or anti-Glγ-giardin morpholino (anti-Glγ-giardin mph; Lane 2). Extracts of these cells were reacted with anti-Glγ-giardin or anti-GlPDI1 antibodies. Abundance of each immunoreactive protein was quantified by densitometry and normalized to that of *Giardia* treated with control morpholino. **b** Expression of Glγ-giardin was monitored by IFAs. Cells were reacted overnight with rat anti-Glγ-giardin polyclonal antibodies (1:100) then treated with Alexafluor 555-conjugated anti-rat IgG (1:100). Slides were mounted with VECTASHIELD anti-fade mounting medium with DAPI then observed with an Axiovert 200 fluorescent microscope. **c** Effect of anti-Glγ-giardin morpholino on cytokinesis of *Giardia* cells. Cells were stained with 10% Giemsa solution and observed with a light microscope to count numbers of cells in interphase (gray columns), mitosis (closed columns) and cytokinesis (open columns). **d** Cell number was determined by counting a least 500 cells per each condition. Cells were attached on coverslips and mounted in anti-fade mounting medium with DAPI. To determine numbers of cells with four or two nuclei, more than 300 cells per condition were counted. *Scale-bars*: **b**, **c**, **d**, 2 µm

To define the role of Glγ-giardin in *G. lamblia*, we designed an anti-Glγ-giardin morpholino to block the translation of Glγ-giardin mRNAs (Table 1). A control morpholino (non-specific oligomers) was also made and transfected into *G. lamblia* WB trophozoites by electroporation (Table 1). These extracts were examined to determine their intracellular levels of Glγ-giardin at 48 h post-transfection by western blot using anti-Glγ-giardin antibodies (Fig. 4a). Cells treated with anti-Glγ-giardin morpholino demonstrated decreases in the amount of Glγ-giardin at 48 h post-transfection to 47% of that of the cells treated with the control morpholino. IFA images using anti-Glγ-giardin antibodies indicated that cells treated with anti-Glγ-giardin morpholino had less fluorescence in the adhesive disc compared with control cells. This result demonstrated the decreased expression of Glγ-giardin in the knockdown cells (Fig. 4b).

The effect of Glγ-giardin knockdown on cell division was determined by Giemsa staining of these cells to distinguish *Giardia* at different stages (i.e. interphase, mitosis and cytokinesis) (Fig. 4c). A small proportion of the cells were in mitosis (1–2%) or cytokinesis (3–5%), while the majority of cells were in interphase (94–95%). Percentages of these cells at different stages were maintained in a similar pattern between control cells and Glγ-giardin knockdown cells. In contrast, an additional assay to determine the mitotic index showed that the proportion of cells with four nuclei increased from 4% of the cells treated with the control morpholino to 10% in cells treated with anti-Glγ-giardin morpholino (Fig. 4d; *P* = 0.003).

### Effect of Glγ-giardin knockdown on formation and function of the ventral disc of *Giardia*

**Fig. 5:**
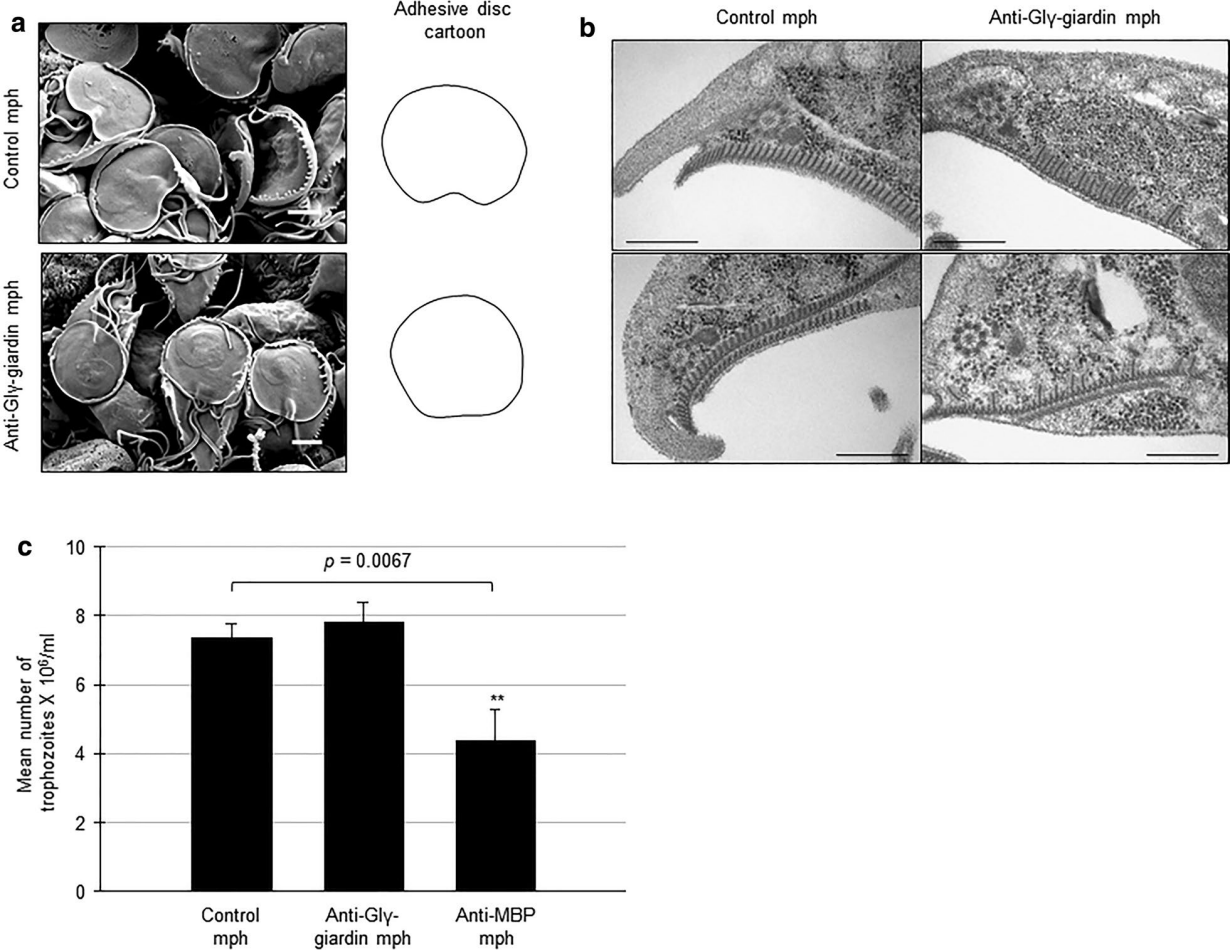
Effect of morpholino-mediated knockdown of Glγ-giardin on structure and function of adhesive disc in *Giardia*. *Giardia* trophozoites were collected 48 h after electroporation with control morpholino, or anti-Glγ-giardin morpholino. **a** Scanning electron microgram showing adhesive disc of *G. lamblia*. Shape of the adhesive discs are presented as cartoons. **b** Transmission electron micrographs demonstrating microribbons of adhesive discs. **c** After 48-h cultivation, culture media were discarded to remove non-attached cells and replaced with PBS. Tubes were incubated on ice for 20 min to detach adherent cells. Detached cells were counted using hemocytometer. As a control, adherence of *G. lamblia* trophozoites transfected with anti-GlMBP morpholino was evaluated as described above. The experiment was performed three times, and three replicates were analyzed for each experiment. The error bars are from three independent experiments. *Scale-bars*: **a**, **b**, 2 µm

*Giardia* cells treated with the control morpholino, or the anti-Glγ-giardin morpholino, were observed by SEM to determine the structural integrity of the adhesive disc (Fig. 5a). The overall structure of the ventral disc appeared intact. However, flattened margins, particularly the ventral groove, were more frequently found in cells treated with anti-Glγ-giardin morpholino. The ventral discs were also observed using TEM (Fig. 5b). When cells were cut in transverse sections, the adhesive discs were observed as a periodical array of filaments connected with MTs in *Giardia* trophozoites treated with control morpholino. On the other hand, the transverse TEM images of *Giardia* treated with anti-Glγ-giardin morpholino clearly demonstrated disruption of the periodical array of filaments in the ventral discs. That is, the portion of the filaments was shortened in the anti-Glγ-giardin morpholino-treated cells.

As a result of the Glγ-giardin knockdown, the shape of the adhesive disc was changed; thus, the following experiment was performed to determine whether the adherence was also affected (Fig. 5c). An additional morpholino was made against *G. lamblia* median body protein (GlMBP; GiardiaDB ORF no. GL50803_16343), which had been previously reported to be involved in adhesion of *Giardia*, and used as a positive control for *Giardia* adherence ability [26]. As expected, treatment with anti-GlMBP morpholino resulted in a 40% reduction of *Giardia* adherence (*P* = 0.0067). In contrast, treatment of *Giardia* with anti-Glγ-giardin morpholino did not induce any change in adherence, indicating that Glγ-giardin may be a structural component of the adhesive disc, but is not a key factor in adherence.

### Expression and localization of Glγ-giardin during encystation

**Fig. 6:**
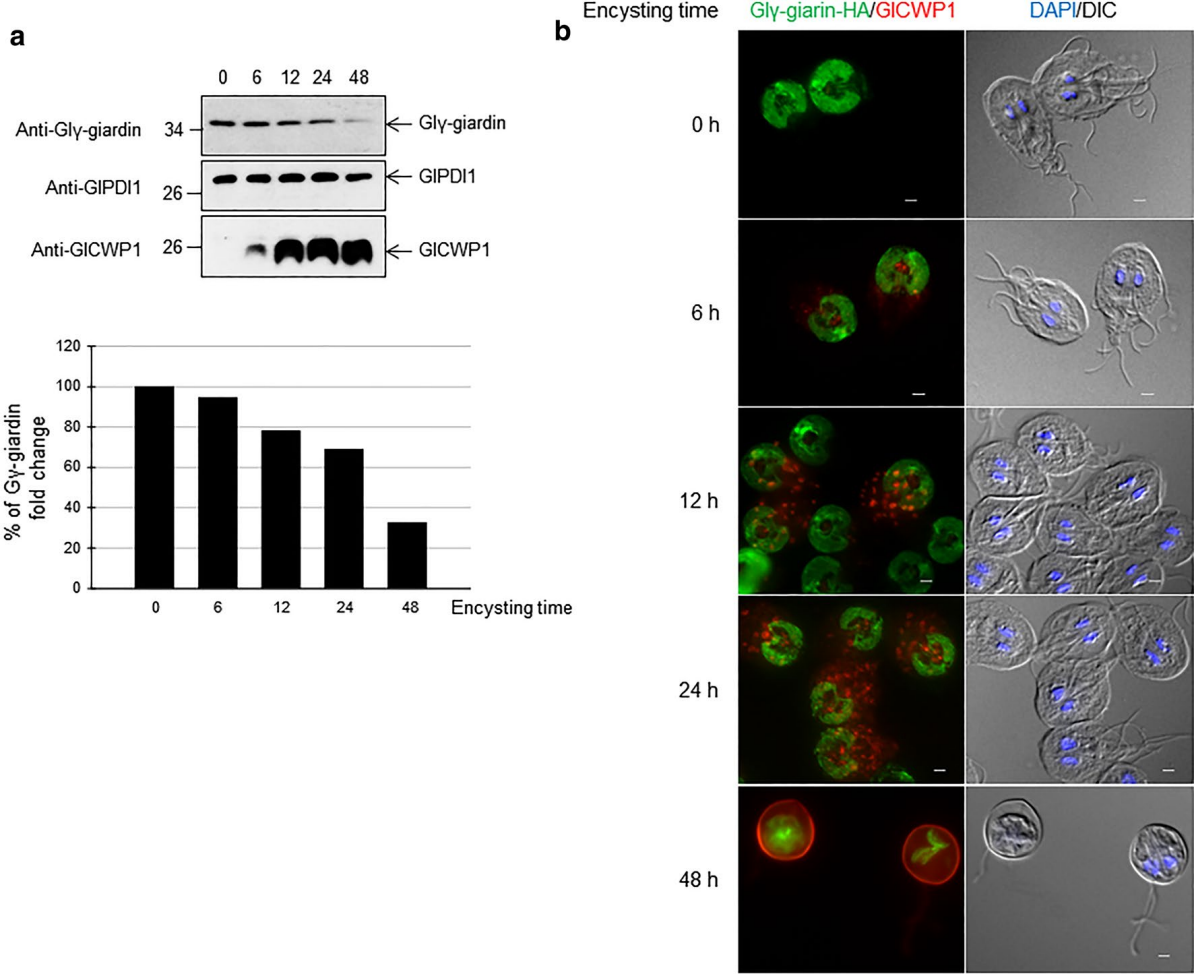
Expression pattern of Glγ-giardin in *G. lamblia* during encystation. **a** Intracellular level of Glγ-giardin during *G. lamblia* encystation was performed by western blot analysis. *Giardia* extracts were prepared at various time-points in trophozoites (0) and encysting cells (6, 12, 24 and 48 h post-induction to encystation). Immunoreactive bands were observed at 35 or 21 kDa, expected sizes of Glγ-giardin or GlCWP1, respectively. After deprobing antibodies with stripping buffer, membrane was incubated with polyclonal rat antibodies specific to PDI1 of *G. lamblia* (1:10,000) as loading control. An immunoreactive band was detected at 26 kDa. Relative expression of Glγ-giardin during encystation is presented as a bar graph. **b** Localization of Glγ-giardin in HA-tagged Glγ-giardin expressing *G. lamblia* during encystation. *Giardia lamblia* attached to glass slides were reacted overnight with mouse anti-HA (1:100) and rat anti-GlCWP1 polyclonal antibodies (1:400) then incubated with Alexafluor 488-conjugated anti-mouse IgG (1:100) and Alexafluor 555-conjugated anti-rat IgG (1:200). Cells were mounted with DAPI containing anti-fade mounting medium and observed. DIC image was acquired to show cell morphology. *Scale-bars*: 2 μm

Since Glγ-giardin was identified as one of the proteins upregulated during the G2-phase, we examined its expression pattern during encystation (Fig. 6a). *Giardia* extracts at various time points of encystation were evaluated for GlCWP1, GlPDI1, and Glγ-giardin. The intracellular level of GlPDI1 served as a loading control. As expected, expression of GlCWP1 was dramatically increased in the encysting cells. Conversely, the intracellular quantity of Glγ-giardin in encysting cells was decreased to 36% of that found in trophozoites.

The intracellular location of Glγ-giardin was examined by IFA using anti-HA antibodies and anti-GlCWP1 antibodies (Fig. 6b). As expected, expression of GlCWP1 started to be detected from the cells at 6 h post-encystation, and increased dramatically to 48 h. Localization of GlCWP in the encystation specific vesicles was clearly demonstrated in encysting cells at 12 h. In the cells at 48 h post-induction, GlCWP was found on the surface of the cells, i.e. cyst wall. Glγ-giardin was mainly observed at the ventral disc in trophozoites. As the encystation process occurred, Glγ-giardin was mainly found in the traces of the adhesive disc of *G. lamblia*.

## Discussion

*Giardia lamblia* is a protozoan, which has a cell cycle composed of two forms, dividing trophozoite and infective cyst. *Giardia* trophozoites have two identical nuclei, both of which are transcriptionally active [34]. Most trophozoites are present at the G2-phase, which is a restriction point determining the pathway for binary division or for differentiation to cyst [35, 36]. Only limited information is available regarding the cell cycle control of *G. lamblia*. Several studies have been conducted to identify cell cycle regulators in *Giardia* differentiation conditions to control the process of *Giardia* division. Reiner et al. [3] demonstrated synchronization of *Giardia* trophozoites using aphidicolin, and monitored the expression of well-known stage-specific genes. Expression of the cyclin B gene was found to be maximal at the G2-phase, whereas expression of the histone genes was upregulated at the S-phase [3]. An additional study using a counterflow centrifugal elutriation method indicated that several well-known cell stage genes (thymidine kinase, minichromosome maintenance 5, polo-like kinase, and proliferating cell nuclear antigen) were upregulated in G2-phase cells [5]. Several investigations on encystation using both transcriptomics [37–39] and proteomics [40–42] resulted in identification of the following as upregulated proteins: metabolic, secretory, trafficking, cytoskeletal-related, transcription factor, variant-specific and hypothetical. To identify a new cell stage-specific protein in *Giardia*, we used aphidicolin to synchronize trophozoites to prepare G1/S-phase and G2-phase cells. Comparative proteomic analysis of G1/S-phase *versus* G2-phase extended the list of these cell stage-specific proteins in *G. lamblia* (Fig. 1 and Table 3). Disappointingly, the majority of the identified genes were categorized as metabolic and hypothetical proteins, for which it is hard to conjecture a relationship with the cell cycle control of *Giardia* trophozoites.

Among the identified proteins showing upregulation at G2-phase, Glγ-giardin, a known component of the ventral disc, was selected for further investigation to monitor the structural change of the ventral disc during the cell cycle. Upregulation of Glγ-giardin at G2-phase was confirmed by two different methods, western blot and quantitative RT-PCR (Fig. 3). IFA indicated that Glγ-giardin was localized at the ventral disc (Fig. 2b), as reported in previous studies [6, 43]. Interestingly, Glγ-giardin was also found in the MB in 12% of interphase trophozoites (data not shown) and localization of Glγ-giardin in MB varied in a phase-dependent manner. That is, more G2-phase cells showed MB labelled with anti-Glγ-giardin antibodies than G1/S-phase cells (Fig. 2c). The MB is a unique cytoskeletal structure hypothesized as a dynamic reservoir of MTs [26, 44]. Recent studies have suggested that the volume of MBs varies depending on the cell stage; this volume gradually increased from G1-phase to G2-phase [5], and the MB has been shown to contribute to the biogenesis of the ventral disc and mitotic spindle, as well as the flagella [45]. In addition, GlMBP isolated from ventral disc was localized in both the ventral disc and MB and played a role in disc biogenesis [26].

Knockdown of expression of Glγ-giardin was performed in trophozoites, and the effect of Glγ-giardin knockdown on cell division was monitored (Fig. 4c, d). Two different assays demonstrated distinct results for cells arrested at cytokinesis, i.e. Giemsa-stained cells and DAPI-stained cells with a less and more dramatic difference, respectively. Despite this difference, knockdown of Glγ-giardin expression resulted in defects in the cell cycle progression of *G. lamblia*.

In addition, *G. lamblia* with lower level of Glγ-giardin was examined with respect to organization and function of the ventral disc (Fig. 5). The effect of Glγ-giardin knockdown on the surface of the ventral disc was not obvious in SEM images (Fig. 5a). Deformation was only found in the ventral groove, a posterior region of the ventral disc with convex curvature, in Glγ-giardin knockdown cells. It was obvious in transverse TEM images of *Giardia* expressing less Glγ-giardin that the formation of microribbons was affected. Shortening of the microribbon occurring uniformly only in daughter cells during division of *Giardia* trophozoites had been previously reported [21]. The *Giardia* ventral disc is composed of MTs, trilaminar microribbons, microribbon-connecting crossbridges, and MT-associated sidearm structures [8, 16, 17, 43]. Recent analysis using negative staining electron tomography and cryo-electron tomography suggested the existence of different functions in different regions of the ventral disc [46]. Glγ-giardin is a unique protein that has no homology with other eukaryotic proteins [6] and has not yet been shown to function. The shortening of microribbon structures and flatting of the ventral groove suggest that Glγ-giardin will play a role in this region of the ventral disc. In our previous research, we found Glγ-giardin that binds to the *G. lamblia* EB1 (GlEB1) protein, a well-known MT-binding protein, by yeast two-hybrid assay using GlEB1 bait [47]. This finding is presumed to support the assumption that various proteins are present in the ventral groove and that these will play a role in curvature of the ventral disc [43, 48]. Further investigation is required to examine how the Glγ-giardin and GlEB1 proteins interactions are involved in the biogenesis of the ventral disc.

Despite the flattening of the ventral groove in *Giardia* with lower levels of Glγ-giardin, there was no defect in the adherence ability of *Giardia* trophozoites (Fig. 5c). The adherence assay appeared to be properly performed, as we observed the knockdown effect of GlMBP [26]. It has been reported that the lateral crest and the bare area play more important roles in attachment than the ventral groove [12, 26].

Using Glγ-giardin as a marker, we monitored the fate of the ventral disc during encystation (Fig. 6). Because ventral disc formation may occur in newly generated trophozoites, we expected lower expression of Glγ-giardin in the cyst stage. Western blot analysis clearly indicated decreased expression of Glγ-giardin during encystation. This result is consistent with the finding of previous studies that transcription of Glγ-giardin was decreased during encystation [19]. Decreased expression of α2-, β-, and δ-giardin was reported in the encysting *G. lamblia* [49] whereas no information is available for the expression of Glγ-giardin during encystation. Therefore, our study extends the list of giardins of which expression was diminished during encystation. IFA indicated that Glγ-giardin was mainly localized to the trail structure of the adhesive disc of the encysting cells (Fig. 6b). During encystation, the ventral disc components have generally been found to be broken down and shrunken [49, 50].

## Conclusions

In the present study, we searched for new proteins related to *Giardia* differentiation through preparation of cell stage-specific cells using aphidicolin, and analyzed the lysate using LC–MS/MS. Glγ-giardin was identified as an upregulated protein in the G2-phase of the *Giardia* cell cycle and a downregulated protein during encystation. Knockdown experiments demonstrated that Glγ-giardin is a component of the trilaminar structure of the ventral disc, which is important in cell division of *G. lamblia*.

## Additional file

**Additional file 1: Figure S1.** Determination of the specificity of the polyclonal antibodies against recombinant *Giardia lamblia* cyclin B protein. **a** Western blot analysis of *Escherichia coli* lysates expressing recombinant Glcyclin B using rat anti-Glcyclin B polyclonal antibodies. **b** Western blot analysis of *Giardia* extracts using rat anti-Glcyclin B polyclonal antibodies. The immunoreactive recombinant Glcyclin B or native Glcyclin B are indicated with arrows.

## Data Availability

The data supporting the conclusions of this article are included within the article and its additional file.

## Abbreviations

Glγ-giardin: *Giardia lamblia* γ-giardin
MT: microtubule
Q-TOF: quadrupole time-of-flight
LC–MS/MS: liquid chromatography–tandem mass spectrometry/mass spectrometry
IFA: immunofluorescence assay
DAPI: 4′,6-diamidino-2-phenylindole
SEM: scanning electron microscopy
TEM: transmission electron microscopy
CWP1: cyst wall protein 1
2-DGE: 2-dimensional gel electrophoresis
MBP: median body binding protein
MB: median body
GlEB1: *Giardia lamblia* EB1
