# Supplementary figures and images for: Role of gamma-giardin in ventral disc formation of Giardia lamblia
Source: Parasit Vectors. 2019 May 14;12:227. doi: 10.1186/s13071-019-3478-8 (PMC6515615; doi:10.1186/s13071-019-3478-8)

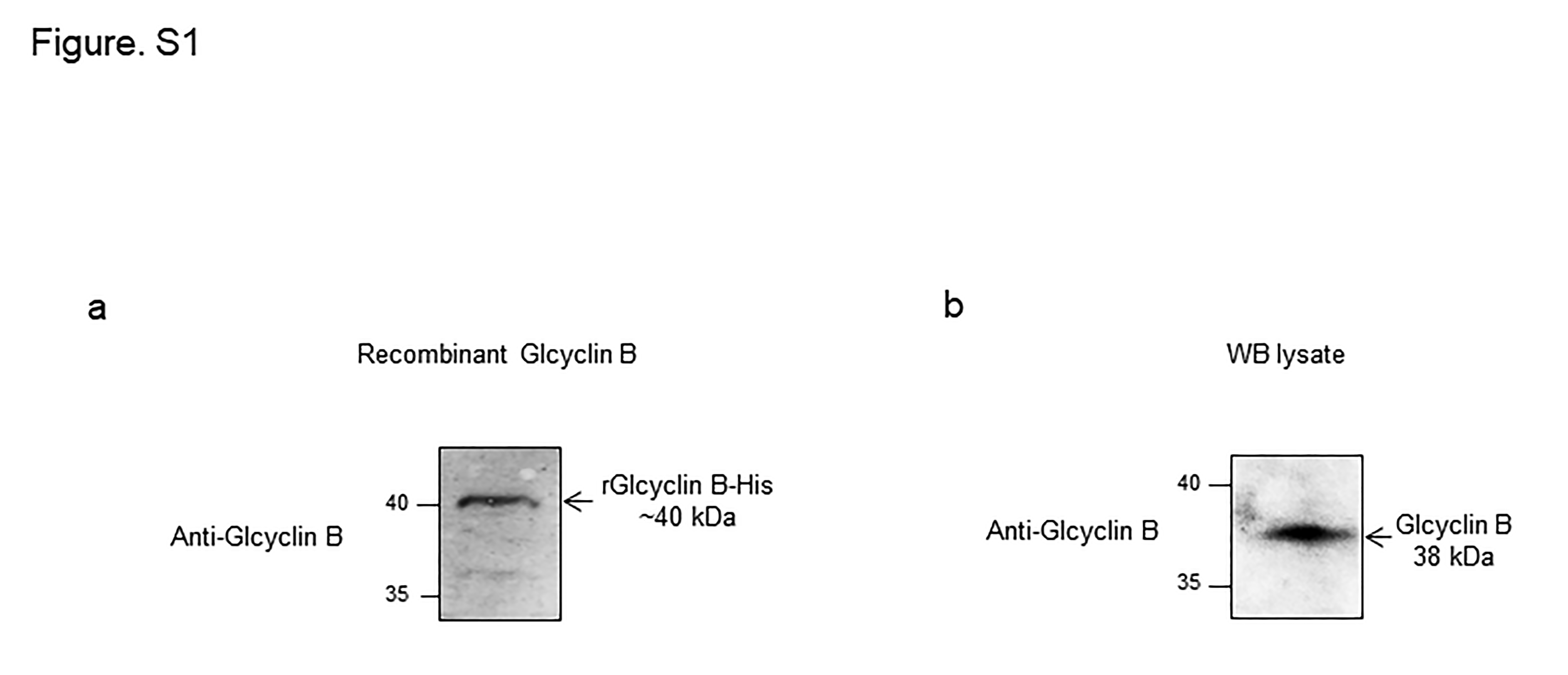

Supplement: Supplementary file 1 — Additional file 1: Figure S1. Determination of the specificity of the polyclonal antibodies against recombinant Giardia lamblia cyclin B protein. a Western blot analysis of Escherichia coli lysates expressing recombinant Glcyclin B using rat anti-Glcyclin B polyclonal antibodies. b Western blot analysis of Giardia extracts using rat anti-Glcyclin B polyclonal antibodies. The immunoreactive recombinant Glcyclin B or native Glcyclin B are indicated with arrows. [file 13071_2019_3478_MOESM1_ESM.tif]
